# A_2A_ Adenosine Receptor Antagonism Reverts the Blood-Brain Barrier Dysfunction Induced by Sleep Restriction

**DOI:** 10.1371/journal.pone.0167236

**Published:** 2016-11-28

**Authors:** Gabriela Hurtado-Alvarado, Emilio Domínguez-Salazar, Javier Velázquez-Moctezuma, Beatriz Gómez-González

**Affiliations:** 1 Area of Neurosciences, Department of Biology of Reproduction, CBS, Universidad Autónoma Metropolitana, Unidad Iztapalapa, Mexico City, Mexico; 2 Postgraduate Program in Experimental Biology, CBS, Universidad Autónoma Metropolitana, Unidad Iztapalapa, Mexico City, Mexico; Emory University School of Medicine, UNITED STATES

## Abstract

Chronic sleep restriction induces blood-brain barrier disruption and increases pro-inflammatory mediators in rodents. Those inflammatory mediators may modulate the blood-brain barrier and constitute a link between sleep loss and blood-brain barrier physiology. We propose that adenosine action on its A_2A_ receptor may be modulating the blood-brain barrier dynamics in sleep-restricted rats. We administrated a selective A_2A_ adenosine receptor antagonist (SCH58261) in sleep-restricted rats at the 10^th^ day of sleep restriction and evaluated the blood-brain barrier permeability to dextrans coupled to fluorescein (FITC-dextrans) and Evans blue. In addition, we evaluated by western blot the expression of tight junction proteins (claudin-5, occludin, ZO-1), adherens junction protein (E-cadherin), A_2A_ adenosine receptor, adenosine-synthesizing enzyme (CD73), and neuroinflammatory markers (Iba-1 and GFAP) in the cerebral cortex, hippocampus, basal nuclei and cerebellar vermis. Sleep restriction increased blood-brain barrier permeability to FITC-dextrans and Evans blue, and the effect was reverted by the administration of SCH58261 in almost all brain regions, excluding the cerebellum. Sleep restriction increased the expression of A_2A_ adenosine receptor only in the hippocampus and basal nuclei without changing the expression of CD73 in all brain regions. Sleep restriction reduced the expression of tight junction proteins in all brain regions, except in the cerebellum; and SCH58261 restored the levels of tight junction proteins in the cortex, hippocampus and basal nuclei. Finally, sleep restriction induced GFAP and Iba-1 overexpression that was attenuated with the administration of SCH58261. These data suggest that the action of adenosine on its A_2A_ receptor may have a crucial role in blood-brain barrier dysfunction during sleep loss probably by direct modulation of brain endothelial cell permeability or through a mechanism that involves gliosis with subsequent inflammation and increased blood-brain barrier permeability.

## Introduction

Long-term insufficient sleep has adverse effects on overall health and impairments in cognition [1, 2]. Cognitive deficits during sleep loss may be related to alterations in the interface between the periphery and the central nervous system, the blood-brain barrier. This barrier restricts the entry of toxic blood-borne molecules into the brain. In this way, chronic sleep restriction induces blood-brain barrier hyperpermeability to Evans blue and sodium fluorescein, increases pinocytosis, and decreases mRNA expression of tight junction proteins in rodents [3, 4]. Interestingly, after brief sleep opportunity periods of 40 and 120 minutes, the blood-brain barrier permeability to Evans blue in almost all brain regions decreases to basal levels excluding cerebellar vermis [3]. Hence, levels of potential molecules involved in blood-brain barrier disruption during sleep loss may increase during waking and are rapidly cleared during sleep restoration. In the central nervous system the nucleotide adenosine has a crucial role as a modulator of neural transmission, as a sleep inductor [5], and a potent regulator of cerebral blood flow [6]. Adenosine concentration in the basal forebrain, hippocampus and cortex increases progressively with prolonged waking [7] or during sleep deprivation, and decreases during sleep [8].

High levels of circulating adenosine or adenosine accumulated in the extracellular space increase the permeability of microvascular endothelial cells that form the blood-brain barrier by its action on A_1_ and A_2A_ adenosine receptors [9–12]. General agonists of adenosine receptors increase 3-fold the blood-brain barrier permeability to 70-kDa dextran coupled to fluorescein (FITC-dextran); however, the administration of a selective A_2A_ receptor agonist (Lexiscan) increases 20-fold the blood-brain barrier permeability to the above mentioned tracer [10]. Selective A_2A_ receptor antagonists are recently used in the treatment of several conditions such as ischemia [13], Parkinson’s disease [14], and multiple sclerosis [9, 10]; in those diseases they are deemed as neuroprotectors by their ability to reduce neuroinflammation. In the present study, we evaluated the effect of an unselective adenosine receptor antagonist (caffeine) and a selective A_2A_ receptor antagonist (SCH58261) on blood-brain barrier integrity in the cortex, hippocampus, basal nuclei and cerebellar vermis of sleep-restricted rats.

## Materials and Methods

### Animals

Three month-old male Wistar rats (n = 71: FITC-Dextrans assay n = 32; Evans blue assay n = 9; Western blot assay n = 27, Immunohistochemistry n = 3) were used. Rats were caged in groups of 4–8 in our laboratory vivarium under a 12-hour light/dark cycle (lights on at 11 pm), at room temperature of 20–25°C. Commercial rat chow and tap water were available *ad libitum* to all rats throughout the experiment. Rats were randomly assigned to the experimental conditions. Care was taken to reduce to the minimum the stress and discomfort in the experimental animals as well as to optimize the number of animals used in the experiments described. Experiments were performed following the Guidelines for the Care and Use of Mammals in Neuroscience and Behavioral Research (National Research Council, 2010) and with the ARRIVE (Animal Research: Reporting In Vivo Experiments) guidelines (www.nc3rs.org.uk/arrive-guidelines) and were approved by the Academic Ethic Committee of the Biological Sciences Division of the Universidad Autonoma Metropolitana, Unidad Iztapalapa.

### Sleep Restriction

For the sleep restriction procedure an acrylic water tank (82cm x 59cm x 48cm) and 7cm diameter platforms were used. Sleep restriction was performed by the multiple platform technique, which abolishes rapid eye movement (REM) sleep and decreases 30% of non-REM sleep, by placing rats over small platforms surrounded by water as previously reported [3]. Rats were kept in the conditions of the multiple platform technique during 20 hours for 10 consecutive days; every day they were allowed to sleep 4 hours in their home-cages during the last 4 hours of the light phase. Intact controls slept *ad libitum* in their home-cages during the 10 days of the experiment.

### Administration of drugs

To evaluate the effect of an unselective adenosine receptor antagonist in sleep-restricted rats caffeine (Sigma C0750) was used; caffeine was dissolved in saline solution and administrated ip at a unique dose of 0.3mg/kg of body weight at the end of the sleep loss period in the 10^th^ day of sleep restriction. To evaluate the effect of a selective A_2A_ receptor antagonist in sleep restricted rats, SCH58261 (5-amino-7-(2-phenylethyl)-2-(2-furyl)-pyrazolo(4,3-e)-1,2,4-triazolo(1,5-c)-pyrimidine) (Sigma S4568) was used. SCH58261 is a potent A_2A_ adenosine receptor antagonist with good selectivity (48–1561 fold) over A_1_, A_2B_, and A_3_ adenosine receptors, and rapid bioavailability after ip administration [15, 16]. SCH58261 was dissolved in dimethyl sulfoxide (DMSO) and administrated ip at 0.01, 0.1, or 0.5mg/kg of body weight at the end of the sleep loss period in the 10^th^ day of sleep restriction (3 times each 30 minutes). Rats remained in the acrylic tank during the drug administration to prevent sleep and were sacrificed 30 minutes after the last SCH58261 administration.

### Corticosterone quantification

An independent experiment was performed to quantify serum corticosterone concentration as a stress biomarker in the sleep restricted rats. Trunk blood was collected between 9–11 am (at the end of the light phase) from controls sleeping *ad libitum* plus DMSO, from 10-day sleep-restricted rats plus DMSO and from 10-day sleep restricted rats plus SCH58261 (n = 3 *per* group). Blood samples were centrifuged at 3000g/10minutes, supernatant was collected and stored at -80°C until processing. Serum corticosterone was quantified by duplicate using corticosterone Enzyme-Linked ImmunoSorbent Assay (ELISA) kit (Abcam, ab108821) following the protocol suggested by the supplier. The kit has an overall intra-assay CV of 5% and inter-assay of 7.1% with a sensitivity of 0.3 ng/mL.

### Blood-brain barrier permeability assays

#### Quantification of blood-brain barrier permeability to 10-kDa FITC-dextrans

Dextrans labeled with Fluorescein isothiocyanate (FITC) (Sigma, FD10S) were suspended in phosphate buffered saline (PBS) to achieve a concentration of 3mg/ml. In a dose—response experiment, rats were divided into control group plus DMSO (Con, n = 4); sleep restriction plus DMSO (SR, n = 3); sleep restriction plus 0.3mg/kg of caffeine (SR+caffeine, n = 3); sleep restriction plus 0.01mg/kg (x3) of SCH58261 (SR+0.01, n = 3); sleep restriction plus 0.1mg/kg (x3) of SCH58261 (SR+0.1, n = 3); sleep restriction plus 0.5mg/kg (x3) of SCH58261 (SR+0.5, n = 3). Rats were anesthetized with sodium pentobarbital (ip. 0.063 g/kg body weight), and 10-kDa FITC-dextran was administrated ic between 9-11am. A 5 mm thoracic incision was done on the left side of each subject, the heart was partially exposed, and 0.2mL/100g body weight of FITC-dextran was administrated in the left heart ventricle. After 10 minutes of FITC-dextran circulation, rats were perfused during 5 minutes with saline solution (0.9%w/v). The brain was removed and dissected; the concentration of 10-kDa FITC-dextran was calculated in hippocampus, basal nuclei, cerebellar vermis and cerebral cortex. Samples were weighed, homogenized and centrifuged at 13500 rpm/10 minutes. Supernatant was collected and absorbance was obtained in a spectrophotometer (Genesys20, Thermo Spectronic) at 520nm. The concentration of 10-kDa FITC-dextran was calculated using a standard curve. Results are showed as the concentration of FITC-dextran per weight of brain tissue (mg/g).

#### Quantification of blood-brain barrier permeability to 70-kDa FITC-dextrans

Dextrans labeled with FITC (Sigma, FD70S) were suspended in PBS to a concentration of 3mg/ml. Rats were divided into control group plus DMSO (Con, n = 3); sleep restriction plus DMSO (SR, n = 3); sleep restriction plus 0.3mg/kg of caffeine (SR+caffeine n = 4); and sleep restriction plus 0.1mg/kg (x3) of SCH58261 (SR+0.1 n = 3). Seventy-kDa FITC-dextran administration procedure and determination of concentration were performed as described above for 10 kDa FITC-dextran.

#### Quantification of blood-brain barrier permeability to Evans Blue

Rats were sacrificed between 9–11 am. Rats were anesthetized with sodium pentobarbital (ip. 0.063g/kg body weight). Evans blue administration was performed at the end of the 10^th^ day of sleep restriction in the control group plus DMSO (Con, n = 3); sleep restriction plus DMSO (SR, n = 3); and sleep restriction plus 0.1mg/kg (x3) of SCH58261 (SR+0.1 n = 3 per group). Evans blue was administrated as previously described [3]; briefly, 0.2mL/100g body weight of Evans blue was administrated in the left heart ventricle. Evans blue circulated during 10 minutes; at the end of that period subjects were perfused with saline solution (0.9%), followed by 4% paraformaldehyde in 0.1 M saline-phosphate buffer (5 minutes each). Brains were post-fixed 24 hours by immersion in the same fixative at 4°C and hand-sectioned into 2 mm coronal sections. Evans blue-stained slices were photographed without magnification with a digital camera (Panasonic, Lumix). Evans blue extravasation was quantitatively analyzed by measuring mean optical density of cortex, hippocampus, basal nuclei, and cerebellar vermis using ImageJ software. Optical density was quantified using the calibrated optical density step tablet and the Rodbard function provided by ImageJ software [3].

### Western blot

To evaluate the expression of tight and adherens junction proteins and the neuroinflammatory markers glial-fibrilar acidic protein (GFAP) and Iba-1, brains were obtained from control group plus DMSO (Con); sleep restriction plus DMSO (SR); and sleep restriction plus 0.1mg/kg (x3) of SCH58261 (SR+0.1). To determine the expression of A_2A_ AR and CD73, brains were obtained from control group plus DMSO (Con) and sleep restriction plus DMSO (SR). The brain was obtained by decapitation and the cerebral cortex, hippocampus, basal nuclei, and cerebellar vermis were dissected, frozen, and stored at -80°C until processing. Protein concentrations were determined using the Bradford assay (BioRad, 500–0006). Proteins (100μg) were resolved using a denaturing 10% SDS-PAGE electrophoresis and transferred to PVDF membranes. Membranes were blocked with 5% w/v non-fat milk in Tris-buffered saline for 2 hours and incubated overnight at 4°C with occludin (Invitrogen, 40–4700, 1:1000), claudin-5 (Abcam, ab53765, 1:1000), ZO-1 (Invitrogen, 40–2200, 1:1000), E-cadherin (Santa Cruz Biotechnology, sc-21791, 1:1000), GFAP (Abcam, ab4648, 1:1000), Iba-1 (Abcam, ab48004, 1:1000), A_2A_ AR (Abcam, ab3461, 1:1000), CD73 (Abcam, ab175396, 1:500), and GAPDH (Abcam, ab8245, 1:1000) antibodies. PVDF membranes were incubated with horseradish peroxidase-conjugated secondary antibodies and revealed with a chemiluminescence detection system (Amersham, RPN2232). Semi-quantitative analysis was performed using C-Digit program (LI-COR iS image studio. Version 3.1). GAPDH was used for normalization of junction proteins, neuroinflammatory markers, CD73 and A_2A_ AR.

### Immunohistochemistry

To corroborate the possible neuroinflammation associated to sleep restriction an immunohistochemistry to detect the astroglial protein GFAP and the microglial protein Iba-1 was performed in free floating brain sections from the following groups: control plus DMSO, sleep restriction plus DMSO, and sleep restriction plus 0.1mg/kg (x3) of SCH58261 (n = 3). To determine the distribution of the A_2A_ adenosine receptor in the studied brain regions, an immunohistochemistry using the A_2A_ antibody (Abcam, ab3461) was also performed in the control plus DMSO and the sleep restricted plus DMSO groups.

Rats were anesthetized with sodium pentobarbital (ip. 0.063 g/kg body weight) and transcardially perfused with normal saline solution followed by a mixture of 4% paraformaldehyde and 1% glutaraldehyde in 0.1 M PBS. The brains were removed and post-fixed in the same fixative during 48 hours at 4°C, followed by cryoprotection in 30% dextrose solution. Thirty-five μm thick coronal sections containing the hippocampus, basal nuclei, and cerebellum were obtained in a cryostat (Leica, CM1850). Sections were preincubated in 1% H_2_O_2_ for 10 min to block endogenous peroxidase activity. After a brief washing with 1% Triton-PBS, sections were incubated in the GFAP (Abcam, ab4648, 1:2000), Iba-1 (Abcam, ab48004, 1:1500), and A_2A_ adenosine receptor (Abcam, ab3461, 1:2000) antibodies diluted in 1% Triton-Normal goat serum-PBS overnight at 4°C. The sections were then washed and incubated in the secondary antibodies, followed by incubation in avidin-biotin complex (ABC kit, Vector Labs, PK6100). Finally the sections were reacted with the peroxidase substrate diaminobenzidine kit (Vector Labs, SK4100), collected on slides, dehydrated and coverslipped. Representative photomicrographs were obtained with a microscope (Olympus, BX60) using a CCD (MediaCybernetics, Evolution VF) and the Image-Pro Plus software.

### Statistical analysis

All of the groups were compared using two-way analysis of variance (ANOVA), using as between-subjects factor the group and as within-subjects factor the regions of interests. The ANOVA tests were followed by orthogonal contrast codes as a *post hoc* test where appropriate. For the comparisons regarding the effect of sleep restriction on serum corticosterone quantification a *t* test for independent samples was performed. P values <0.05 were considered significant; a *post-hoc* power test was performed for all the ANOVA tests. All values were represented as mean ± standard error of the mean (s.e.m.). Statistical analyses were conducted using JMP software (SAS Institute Inc., version 12.2.0).

## Results

**Fig 1:**
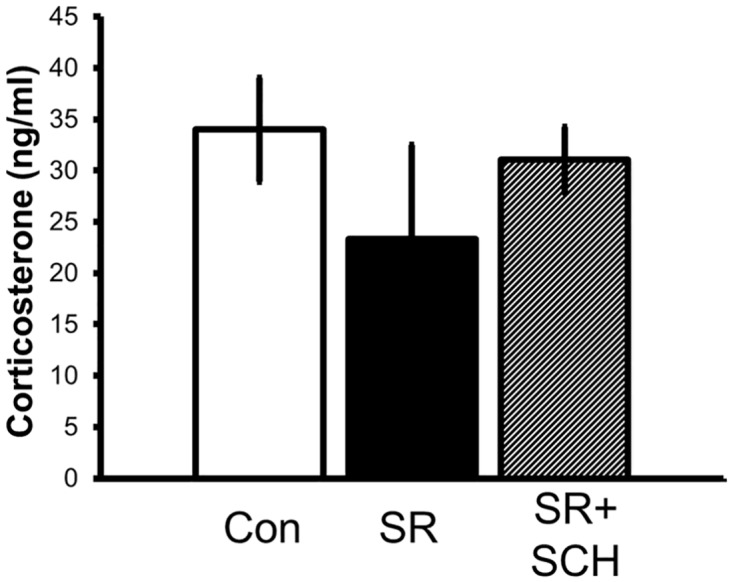
Corticosterone concentration in control and sleep-restricted groups. Control plus vehicle DMSO (Control), sleep restriction plus DMSO (SR), and sleep restriction plus SCH58261 at 0.1mg/kg (SR+SCH) (n = 3 *per* group). Mean ± s.e.m.

As shown in Fig 1 the protocol used to induce sleep restriction did not modify serum corticosterone levels; moreover, SCH58261 did not affect corticosterone levels in the sleep-restricted rats.

### Adenosine receptor antagonism reverted the blood-brain barrier hyperpermeability induced by sleep restriction

**Fig 2:**
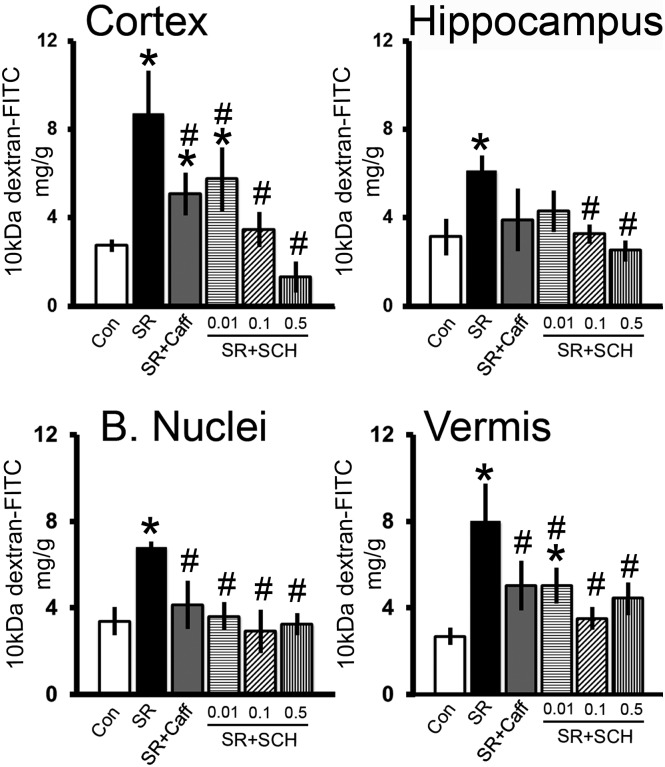
Adenosine receptor antagonism reverts the effect of sleep restriction on blood-brain barrier permeability to 10-kDa FITC-dextran. Graphs show the concentration (mg/g of tissue) of 10-kDa FITC-dextran in the cortex, hippocampus, basal nuclei, and cerebellar vermis of the following groups: control plus DMSO (Con), sleep restriction plus DMSO (SR), sleep restriction plus caffeine at 0.3mg/kg (SR+Caff), sleep restriction plus SCH58261 at 0.01mg/kg (SR+SCH 0.01), sleep restriction plus SCH58261 at 0.1mg/kg (SR+SCH 0.1), and sleep restriction plus SCH58261 at 0.5mg/kg (SR+SCH 0.5) (n = 3–4 per group). Mean ± s.e.m. Two-way ANOVA test, *post hoc* test orthogonal contrast codes, *p<0.05 with respect to the control group. ^#^p<0.05 with respect to the sleep restriction group.

Sleep restriction increased blood-brain barrier permeability to 10-kDa FITC-dextran and adenosine receptor antagonism reverted the effect of sleep restriction in almost all brain regions studied (Group effect, F_5_ = 16.756, p<0.001, Power = 0.869). As shown in Fig 2 sleep restriction increased the concentration of 10-kDa FITC-dextran in the cortex (p<0.001), basal nuclei (p = 0.003), hippocampus (p = 0.010), and vermis (p<0.001) in comparison with the control group. A single dose of caffeine (0.3mg/kg of body weight) decreased the blood-brain barrier hyperpermeability induced by sleep restriction to 10-kDa FITC-dextran in the cortex (p = 0.003), basal nuclei (p = 0.03), and vermis (p = 0.01) (Fig 2). The administration of the A_2A_ selective antagonist, SCH58261, reduced the permeability to 10-kDa FITC-dextran in sleep-restricted rats in a dose response manner in the cortex. The dose of 0.1mg/kg of body weight of SCH58261 had statistical significant differences with respect to sleep restriction group in the cortex (p<0.001), basal nuclei (p = 0.002), hippocampus (p = 0.022), and cerebellar vermis (p<0.001) (Fig 2).

**Fig 3:**
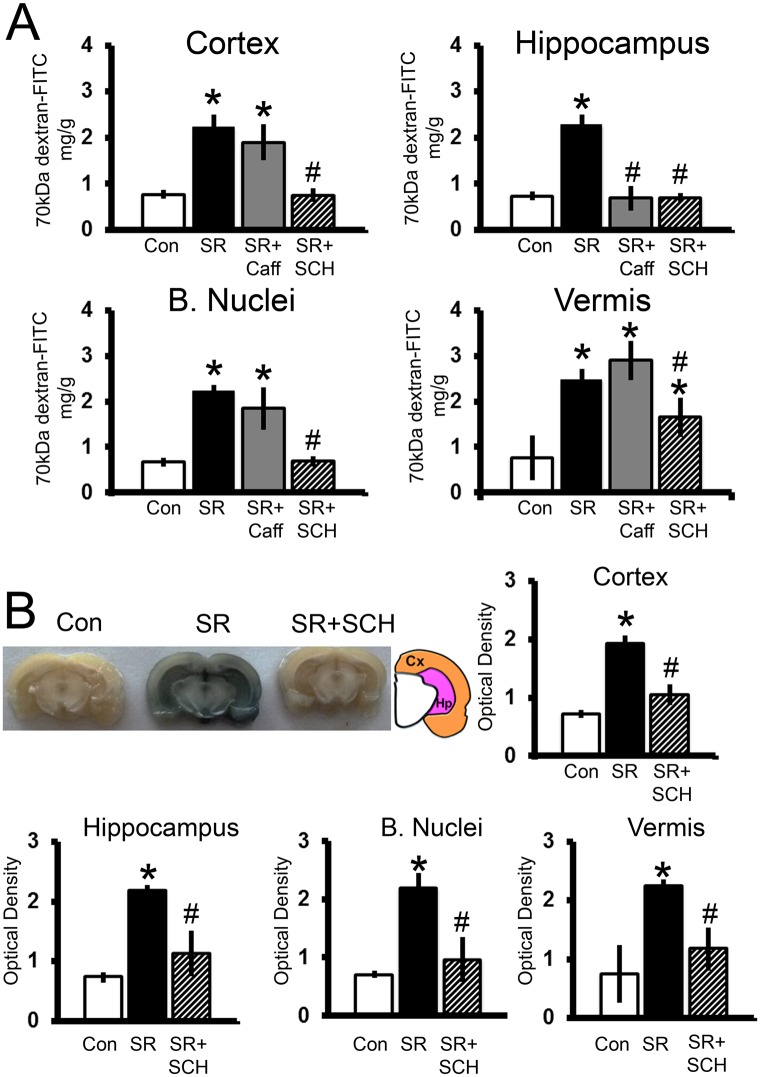
Adenosine receptor antagonism reverts the effect of sleep restriction on blood-brain barrier permeability to 70-kDa FITC-dextran and Evans blue. In A, graphs show the concentration (mg/g of tissue) of 70-kDa FITC-dextran in the cortex, hippocampus, basal nuclei, and vermis of the following groups: control plus vehicle DMSO (Con), sleep restriction plus DMSO (SR), sleep restriction plus caffeine at 0.3mg/kg (SR+Caff), and sleep restriction plus SCH58261 at 0.1mg/kg (SR+SCH). In B are depicted representative coronal slices of Evans blue extravasation in the cortex (Cx) and hippocampus (Hp) of the control (Con), sleep restricted (SR) and sleep restricted plus SCH58261 at 0.1mg/kg (SR+SCH) groups. Quantification of Evans blue deposition is shown as relative units of optical density in the cortex, hippocampus, basal nuclei, and vermis of the following groups: control plus vehicle DMSO (Con), sleep restriction plus DMSO (SR), and sleep restriction plus SCH58261 at 0.1mg/kg (SR+ SCH) (n = 3–4 per group). Mean ± s.e.m. Two-way ANOVA test, *post hoc* test orthogonal contrast codes, *p<0.05 as compared to the control group, ^#^p<0.05 with respect to the sleep restriction group.

Sleep restriction increased blood-brain barrier permeability to 70-kDa FITC-dextran and the antagonism of adenosine receptors reverted the blood-brain barrier hyperpermeability (Group x Region effect, F_3-9_ = 3.409, p = 0.004, Power = 0.958). As shown in Fig 3A, sleep restriction increased the 70-kDa FITC-dextran concentration in the cortex (p<0.001), hippocampus (p<0.001), basal nuclei (p<0.001), and cerebellar vermis (p<0.001) as compared to controls sleeping *ad libitum*. Caffeine treatment reverted the increase of 70-kDa FITC-dextran concentration induced by sleep restriction only in the hippocampus (p<0.001). The administration of SCH58261 (0.1mg/kg (x3)) reverted the increase of the concentration of 70-kDa FITC-dextran induced by sleep restriction in the cortex (p<0.001), hippocampus (p<0.001), basal nuclei (p<0.001), and cerebellum (p = 0.025) (Fig 3A).

Sleep restriction increased the blood-brain barrier permeability to Evans blue and the administration of SCH58261 at 0.1mg/kg (x3) in sleep-restricted rats restored normal blood-brain barrier permeability (Group effect, F_2_ = 56.462, p<0.001, Power = 0.999) (Fig 3A). Sleep restriction increased the blood-brain barrier permeability to Evans blue in all the brain regions (p<0.001) and the administration of SCH58261 at 0.1mg/kg (x3) in sleep-restricted rats restored normal blood-brain barrier permeability to Evans blue in those brain regions (p<0.01) (Fig 3B).

### Selective A_2A_ adenosine receptor antagonism reverted the sleep restriction-dependent decrease in tight junction protein expression

**Fig 4:**
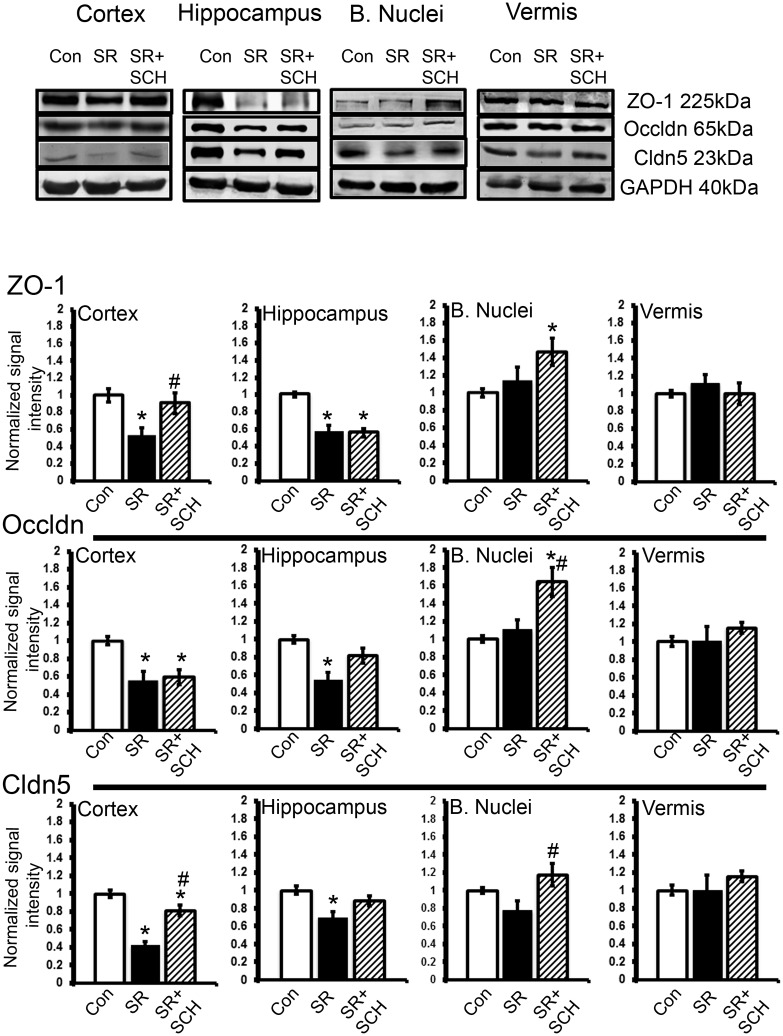
A_2A_ adenosine receptor antagonism reverts the effect of sleep restriction on tight junction protein expression. At the top are depicted representative western blots of tight junction protein expression in the cortex, hippocampus, basal nuclei and vermis. Graphs show the relative optical density of tight junction proteins on the following groups: control plus DMSO (Con), sleep restriction plus DMSO (SR), and sleep restriction plus SCH58261 at 0.1mg/kg (SR+SCH) (n = 6 per group). GAPDH was used for normalization. Abbreviations are as follow: Cldn5: claudin-5; Occldn: occludin; ZO-1: Zonula occludens-1. Mean ± s.e.m. Two-way ANOVA test, *post hoc* test orthogonal contrast codes, *p<0.05 as compared to the control group. ^#^p<0.05 with respect to the sleep restriction group.

Because the main and consistent effect on blood-brain barrier permeability in sleep-restricted rats was observed with the SCH58261 treatment at a dose of 0.1mg/kg, tight junction protein expression experiment was performed only in that group as compared to intact controls and sleep restricted rats treated with the vehicle (DMSO). We found that the relative expression of claudin-5 (23kDa) decreased in the cortex, hippocampus, and basal nuclei of sleep-restricted rats as compared to control rats sleeping *ad libitum* and SCH58261 normalized claudin-5 expression in sleep restricted rats (Group x Region effect, F_2-6_ = 2.792, p = 0.018, Power = 0.896). In comparison to the control group, sleep restriction induced a 50% decrease in the relative expression of claudin-5 in the cortex (p<0.001). SCH58261 induced a recovery of claudin-5 expression in the cortex (p = 0.001) (Fig 4). In the hippocampus the reduction of claudin-5 expression was 30% (p = 0.011), and the administration of SCH58261 restored to basal levels the relative expression of claudin-5 (Fig 4). In the basal nuclei, the relative expression of claudin-5 tend to decrease in the sleep restriction group (approximately 20%), nevertheless we did not find significant differences. In that region, the group of sleep-restricted rats administrated with SCH58261 had statistically significant differences with the sleep restriction group; those SCH58261 treated animals presented an increase in the relative expression of claudin-5 as compared to sleep-restricted rats (p = 0.001) (Fig 4). As shown in Fig 4 no differences were observed in the relative expression of claudin-5 in the vermis under any experimental condition in comparison to the control group.

The relative expression of the tight junction proteins occludin and ZO-1 varied as a function of the region studied in the sleep restricted rats and also in the animals treated with the selective A_2A_ adenosine receptor antagonist SCH58261 (Occludin: Group x Region effect, F_2-6_ = 6.382, p<0.001, Power = 0.997; ZO-1: Group x Region effect, F_2-6_ = 5.551, p<0.001, Power = 0.993). Western blot analysis showed that the relative levels of occludin (65kDa) in the cortex decreased 45% in the sleep-restricted rats as compared to intact controls (p = 0.001) but the treatment with SCH58261 did not restore the basal relative expression of occludin (p = 0.003 as compared to intact controls). In the case of the relative expression of ZO-1 in the cortex, sleep restriction reduced 46% the expression in comparison to the control group (p = 0.001) and the treatment with SCH58261 restored to the basal levels its expression (Fig 4). In the hippocampus, a significant difference in comparison to the control group was detected in the expression of occludin, in which the decrease was 45% (p = 0.001), and ZO-1, with a decrease of 44% (p = 0.002) in the sleep restriction group. The treatment with SCH58261 restored the basal levels of occludin but not of ZO-1 (p = 0.002) (Fig 4). Sleep restriction did not induce significant changes in occludin and ZO-1 relative expression in the basal nuclei, however we found that the treatment with SCH58261 increased 64% the levels of occludin with respect to the control group (p<0.001) and 54% with respect to the sleep-restriction group (p<0.001) (Fig 4). No changes were observed in the expression of occludin and ZO-1 in the cerebellar vermis (Fig 4). Regarding the adherens junction protein E-cadherin, no clear pattern of change was observed in any of the groups; sleep restriction increased E-cadherin protein expression only in the hippocampus and SCH58261 did not modify the levels of the protein in the sleep restricted rats (see S1 Fig).

### SCH58261 attenuates the overexpression of neuroinflammatory markers induced by sleep restriction

**Fig 5:**
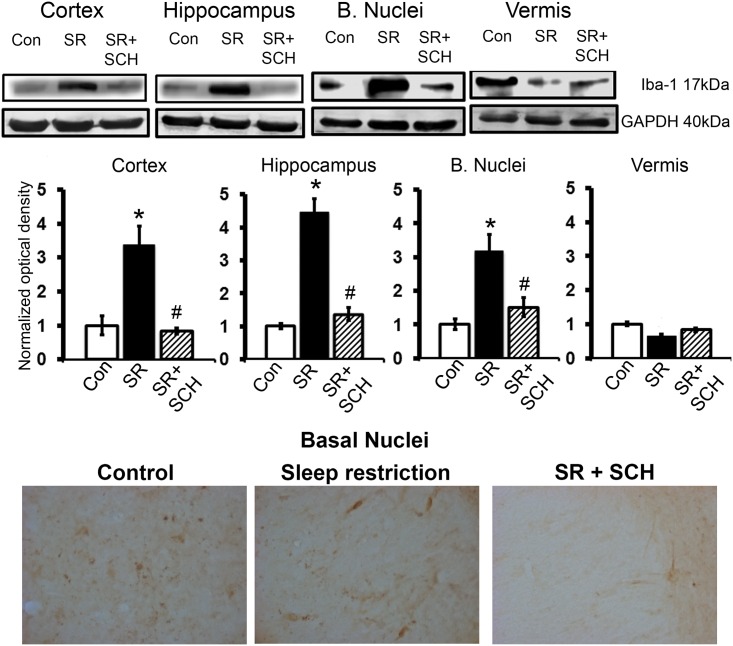
A_2A_ adenosine receptor antagonism attenuates Iba-1 overexpression induced by sleep restriction. Representative western blot images of expression of Iba-1 *per* region (cortex, hippocampus, basal nuclei, and vermis) are shown, as well as graphs depicting the relative optical density of Iba-1 in the following groups: control plus DMSO (Con), sleep restriction plus DMSO (SR), and sleep restriction plus SCH58261 at 0.1mg/kg (SR+SCH) (n = 3 *per* group). GAPDH was used for normalization. Mean ± s.e.m. Two-way ANOVA test, *post hoc* orthogonal contrast codes, *p<0.05 as compared to the control group; ^#^p<0.05 with respect to the sleep restriction group. In the lower panel, microphotographs show the differences in the number of Iba-1 immunoreactive cells in the basal nuclei of control, sleep restricted and sleep restricted plus SCH groups. X400.

As shown in Fig 5, sleep restriction increased the relative expression of Iba-1 (17 kDa), a marker of reactive microglia, in almost all brain regions as compared to control rats sleeping *ad libitum*, and SCH58261 restored to normal levels the Iba-1 expression in sleep restricted rats (Group x Region effect, F_2-6_ = 10.621, p<0.001, Power = 0.862). Sleep restriction increased Iba-1 expression in the cortex (p<0.001), hippocampus (p<0.001), and basal nuclei (p<0.001) (Fig 5), but not in the cerebellar vermis. As shown in the lower panel of Fig 5, Iba-1 overexpression seen in western blot was related to a higher number of Iba-1 immunoreactive cells at least in the basal nuclei.

**Fig 6:**
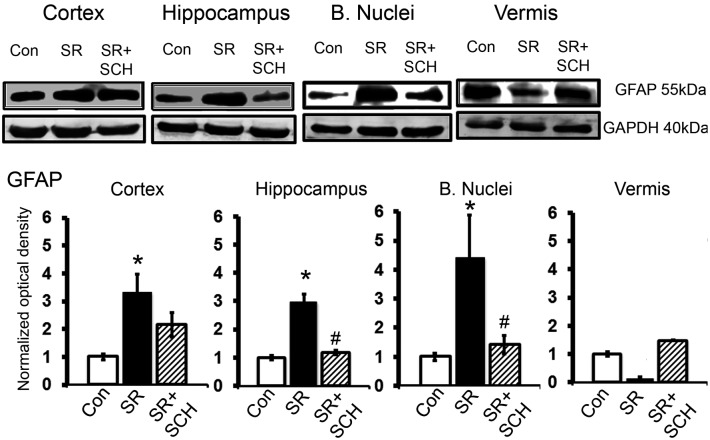
A_2A_ adenosine receptor antagonism attenuates GFAP overexpression induced by sleep restriction. Representative western blot images of expression of GFAP *per* region (cortex, hippocampus, basal nuclei, and vermis) are shown, as well as graphs depicting the relative optical density of GFAP in the following groups: control plus DMSO (Con), sleep restriction plus DMSO (SR), and sleep restriction plus SCH58261 at 0.1mg/kg (SR+SCH) (n = 3 *per* group). GAPDH was used for normalization. Mean ± s.e.m. Two-way ANOVA test, *post hoc* orthogonal contrast codes, *p<0.05 as compared to the control group; ^#^p<0.05 with respect to the sleep restriction group.

**Fig 7:**
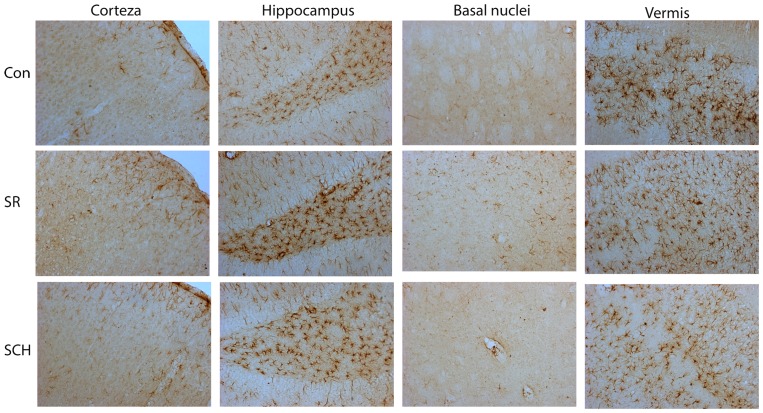
A_2A_ adenosine receptor antagonism reverts the astroglial hyper-ramification induced by sleep restriction. Photomicrographs show the astroglial morphology in the controls plus DMSO, sleep restricted plus DMSO, and sleep restriction plus SCH58261 at 0.1mg/kg in the cortex, hippocampus, basal nuclei, and cerebellar vermis. X100.

In the same way, sleep restriction increased the relative expression of the astroglial marker GFAP (55 kDa), this effect was also reverted with SCH58261 administration (Group x Region effect, F_2-6_ = 4.538, p = 0.003, Power = 0.952) (Figs 6 and 7). Sleep restriction increased GFAP expression in the cortex (p = 0.003), hippocampus (p = 0.010), and basal nuclei (p<0.001), while did not modify GFAP expression in the cerebellar vermis. A_2A_ adenosine receptor antagonism reverted to basal levels the sleep-related GFAP overexpression in all the three regions. As shown in Fig 7 the GFAP changes in sleep restricted rats were related to increased astroglial ramifications rather than apparent changes in astroglial density in the cortex, hippocampus and basal nuclei.

### Sleep restriction modulates the expression of A_2A_ adenosine receptor but not of the adenosine-synthesizing enzyme CD73

**Fig 8:**
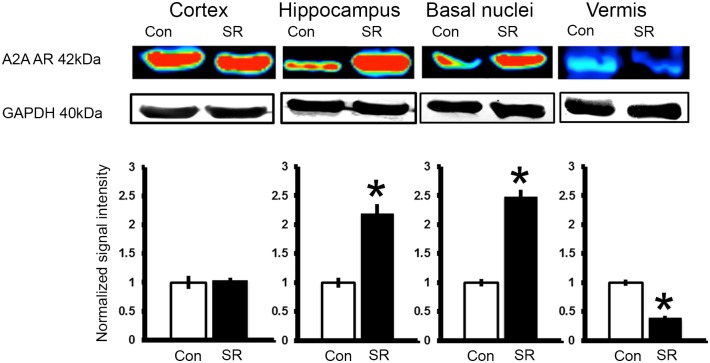
Sleep restriction modulates A_2A_ adenosine receptor expression. At the top, representative western blot of the expression for A_2A_ adenosine receptor in the cortex, hippocampus, basal nuclei, and vermis using chemiluminescent signal. Graphs show the relative optical density of A_2A_ adenosine receptor in the following groups: control plus DMSO (Con) and sleep restriction plus DMSO (SR) (n = 3 *per* group). GAPDH was used for normalization. Mean ± s.e.m. Two-way ANOVA test, *post hoc* orthogonal contrast codes, *p<0.05 as compared to the control group.

**Fig 9:**
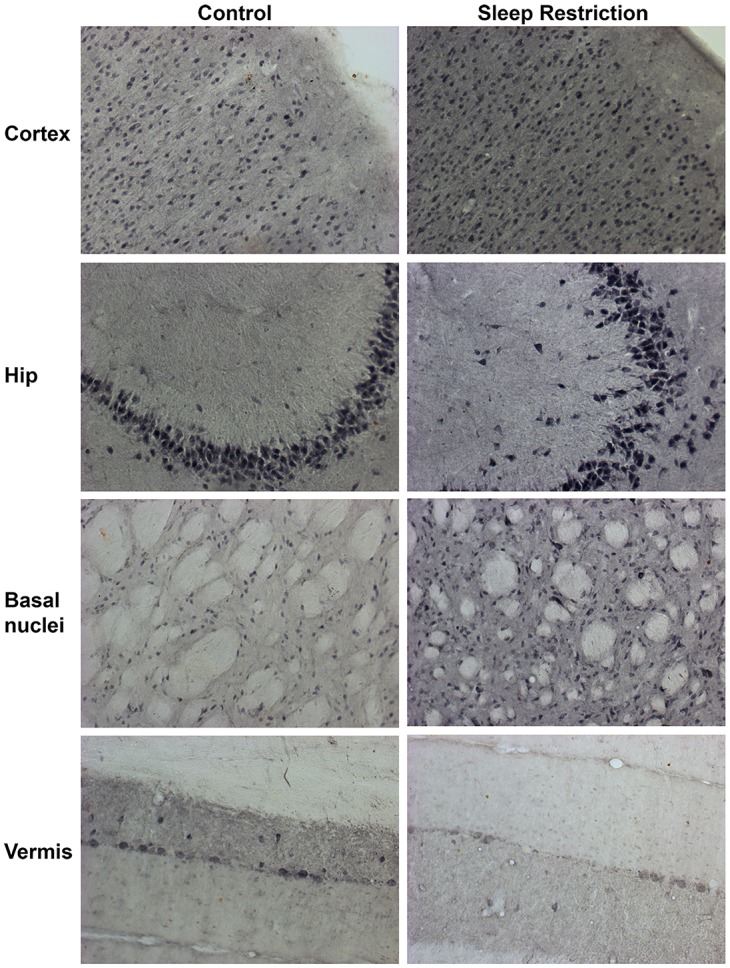
Sleep restriction modifies A_2A_ adenosine receptor expression in brain parenchymal cells. Photomicrographs show the distribution of A_2A_ adenosine receptor in the cortex, hippocampus, basal nuclei, and cerebellar vermis of the following groups: controls plus DMSO, sleep restriction plus DMSO, and sleep restriction plus SCH58261 at 0.1mg/kg. Note the expression of A_2A_ adenosine receptors in cerebellar capillaries. X100.

Because the differential effect of sleep restriction and SCH58261 treatment on blood-brain barrier permeability, tight junction and neuroinflammatory markers expression may be associated to the differential density of A_2A_ adenosine receptors in each brain region; we tested the expression of this receptor in the cortex, hippocampus, basal nuclei, and vermis of sleep-restricted rats and intact controls. We found that sleep restriction modified the A_2A_ adenosine receptor expression in a region dependent way (Group x Region effect, F_1-3_ = 94.724, p<0.001, Power = 0.999) (Figs 8 and 9). In the hippocampus sleep restriction increased 2-fold the expression levels of A_2A_ adenosine receptors as compared to the control group (p<0.001). In the same way, the relative expression of A_2A_ adenosine receptors in the basal nuclei increased 2-fold than the control group (p<0.001); however, in the cerebral cortex sleep restriction did not modify the relative expression of A_2A_ adenosine receptors (as shown in Fig 8). By the contrary, in the cerebellar vermis the relative expression of A_2A_ adenosine receptors decreased 50% in the group of sleep restriction as compared to intact controls (p<0.001) (Fig 8). As shown in Fig 9, A_2A_ adenosine receptors expressed in brain parenchymal cells, mainly in neurons and endothelial cells.

Besides A_2A_ adenosine receptor differential distribution along brain regions, other possible mechanism involved in the differential regional effects of sleep restriction on blood-brain barrier function implied the differential expression of the adenosine-synthesizing enzyme, the ecto-5’-nucleotidase (CD73), in the studied brain regions. As shown in S2 Fig western blot analysis showed that the expression of CD73 remained without change in all brain regions of the sleep restricted rats as compared to controls sleeping *ad libitum*.

## Discussion

Sleep restriction increased blood-brain barrier permeability to FITC-dextran and Evans blue; the changes in blood-brain barrier permeability were observed for both low- and high-molecular weight tracers in the cortex, hippocampus, basal nuclei and cerebellar vermis of sleep-restricted rats as compared to controls sleeping *ad libitum*. The use of an unselective adenosine receptor antagonist, caffeine, reverted the blood-brain barrier hyperpermeability to a low molecular weight tracer in all brain regions, but only partially for large molecules; caffeine reverted the sleep restriction-related hyperpermeability in the hippocampus but failed to do that in the cortex, basal nuclei and cerebellar vermis. Meanwhile, the administration of a selective A_2A_ adenosine receptor antagonist (SCH58261) fully restored the normal permeability of the blood-brain barrier to both low- and high-molecular weight tracers in the cortex, hippocampus and basal nuclei; however, only the dose of 0.1mg/kg had consistent significant differences with respect to the sleep restriction group. The changes in blood-brain barrier permeability were accompanied by corresponding changes in tight junction protein expression; sleep restriction decreased the expression of claudin-5, occludin, and ZO-1 in almost all brain regions studied, while did not affect the expression of the adherens junction protein E-cadherin. The selective antagonism of A_2A_ adenosine receptor was able to revert the changes in tight junction protein expression in sleep-restricted rats. Regional differences in blood-brain barrier permeability associated to chronic sleep loss may have arisen from differences in the expression of A_2A_ adenosine receptors as well as on its selective localization on glial or endothelial cells, because there were not differences among groups in the expression of the adenosine-synthesizing enzyme CD73 in the studied brain regions.

Sleep loss effects on physiology may potentially be confounded with unspecific stress effects associated to the sleep restriction procedures [17], here we reported that sleep restriction during 10 days using the multiple platform technique did not alter the serum corticosterone concentration. Serum corticosterone was quantified at the end of the light phase, near the normal circadian peak of plasma corticosterone, other studies using a similar sleep restriction protocol have shown that rises in corticosterone concentration occur only during the 20 hours of sleep deprivation *per* day since the day 1 until day 7 of sleep restriction [18]. In addition, similar to our findings, previous reports have shown that during the hours of sleep opportunity corticosterone returns to basal levels [17, 18]. The fact that we found low levels of serum corticosterone near the peak of the corticosterone circadian rhythm indicates a potential deregulation of the hypothalamus-pituitary-adrenal axis during sleep loss, therefore more experiments are needed to test potential changes in the rhythmicity of corticosterone associated to chronic sleep loss, as has been shown for chronic stress [19].

Generally, the passage of small molecules (5–40 kDa) from blood-to-brain is considered a marker of blood-brain barrier permeability to small solutes and ions, while Evans blue dye (~65kDa), which binds to albumin, is a marker for protein permeability [20]. Therefore our results indicate that sleep restriction could modify both ion and protein permeability in the blood-brain barrier and that A_2A_ adenosine receptor antagonism reverted both effects. Similar results have been described in animal models of brain diseases in which an initial increase in blood-brain barrier permeability is reverted to basal levels after adenosine receptor antagonism, eg. in experimental autoimmune encephalomyelitis [10].

The role of adenosine receptors in regulating blood-brain barrier permeability is amply described [9–12]. However a recent report showed that stimulation of adenosine receptors modified blood-brain barrier permeability to low molecular weight FITC-dextrans by a mechanism independent of blood-brain barrier regulation *per se*, by increasing FITC-dextrans plasma availability secondary to impaired renal function [21]; those findings impel a thoughtful review of the role of adenosine in regulating blood-brain barrier function. We antagonized physiological levels of adenosine, particularly adenosine acting on A_2A_ receptors; A_2A_ receptors are involved in the maintenance of renal medullary blood-flow through vasodilation and are not involved in fluid or ion reabsorption (reviewed in [22]), therefore it is possible that A_2A_ adenosine receptor antagonism may have had little effect on dextran-FITC clearance from the blood. In addition, the lack of kidney damage during sleep loss [23], which guarantees a normal clearance of FITC-dextrans through urine, and the fact that we used Evans blue, an albumin-binding dye with little renal clearance [21], allow us to affirm that A_2A_ adenosine receptors are playing a central role in regulating blood-brain barrier permeability during sleep loss by directly acting on blood-brain barrier cellular components (eg. endothelial cells, pericytes, and astrocytes) or on microglial cells.

It is known that adenosine receptors are amply distributed in the rat brain; particularly A_2A_ receptor is highly expressed in the basal nuclei, hippocampus and cortex, but its expression is low in the cerebellum [24, 25]; A_2B_ and A_3_ adenosine receptors are poorly expressed in the brain, and A_1_ adenosine receptor is highly expressed in the cerebellum and widespread distributed in the rest of the brain but with low levels [24]. In this way, the overexpression of A_2A_ adenosine receptor in the hippocampus of sleep restricted rats but not in the cortex, basal nuclei or vermis, explains the reduction of hyperpermeability to 70-kDa FITC-dextran by caffeine administration in the hippocampus but not in other brain regions. These data agree with a previous report that showed that sleep restriction in mice increase the mRNA levels of A_2A_ adenosine receptor in the hippocampus but not in other brain regions [26]. The hippocampus is predominantly affected by insufficient sleep; hence, sleep loss negatively impacts memory, causing deficits in memory processes, which can be attenuated with caffeine administration [27]. Therefore, caffeine is considered as a potent neuroprotector and this property has been associated to caffeine action on A_2A_ adenosine receptor [28], then it is very likely that the effect of caffeine on the restoration of normal blood-brain barrier permeability after sleep restriction is mediated by A_2A_ adenosine receptor.

The role of A_2A_ adenosine receptor in regulating the blood-brain barrier during sleep loss is supported by the fact that the used antagonist, SCH58261, is 48, 581, and 1561 fold more selective to A_2A_ adenosine receptor than to A_1_, A_2B_, and A_3_ adenosine receptors, respectively [15, 16]. Moreover, the low dose used in this experiment is similar to the dose used in previous *in vivo* studies that showed that SCH58261 neuroprotective effects are only observed at very low doses (in the range of 0.01–0.5 mg/kg) [29]. The fact that SCH58261 modified blood-brain barrier permeability only in the brain regions enriched in A_2A_ adenosine receptors pinpoints to a regional highly selective regulation of blood-brain barrier function during sleep loss and recovery by adenosine acting on its A_2A_ receptors.

Regarding the mechanism by which sleep loss increases blood-brain barrier permeability, here, we report that sleep restriction decreased the expression of tight junction proteins, including claudin-5, ZO-1, and occludin in the hippocampus and cortex. The decrease in the expression of these proteins is related to the induction of hyperpermeability in brain endothelial cells under pathological conditions [30]. Claudin-5 is necessary for endothelial barrier integrity, it has been reported that in human brain endothelial cells, a selective A_2A_ adenosine receptor agonist (Lexiscan) decreased gradually the expression of claudin-5 up to 30 minutes after treatment; meanwhile, the treatment with an unselective adenosine receptor agonist decreased the expression of claudin-5 after 2 hours post treatment [11]. This supports our hypothesis that the rapid action of adenosine and particularly its action on A_2A_ adenosine receptors might regulate the blood-brain barrier permeability in sleep-restricted rats. Indeed, we found that brief sleep opportunity periods (40–120 minutes) induced a recovery of claudin-5 expression (unpublished data) suggesting that the degradation of adenosine during sleep recovery prevents the A_2A_ adenosine receptor activation restoring the blood-brain barrier integrity. In this way, blocking A_2A_ adenosine receptor in sleep-restricted rats may promote the physiological expression of claudin-5 and therefore recover the basal permeability of the blood-brain barrier.

The decrease in the expression of ZO-1 and occludin is maintained even with SCH58261 administration, suggesting that other pathways are yet activated. For instance, one hallmark characteristic of sleep loss is the generation of a low-grade inflammatory status (reviewed in [31]). Several inflammatory mediators, such as the cytokines tumor necrosis factor (TFN)-α, interleukin (IL)-1β, IL-6, and IL-17A [32] as well as molecules such as C-reactive protein (CRP), cyclooxygenase (COX)-2, and endothelin (ET)-1 [4] are released during sleep loss and are able to modify tight junction protein expression both in *in vitro* and in *in vivo* experiments (for a review see [33, 34]). In addition, the decrease of ZO-1 and occludin might be associated to subtle permeability changes related to peripheral inflammation like that occurring in naturally aged rodents [35].

The maintenance of relative expression of tight junction proteins in the basal nuclei suggest that in this region the mechanism of blood-brain barrier dysfunction might be mediated by other unselective transport such as pinocytosis or that these proteins are present but have a different localization, for example in the cytoplasm. Because basal nuclei have more A_2A_ adenosine receptors than other brain regions, we also propose that the possible re-distribution of tight-junction proteins might be restored after SCH58261 administration, indeed, we observed that levels of claudin-5 and occludin increased with the administration of this antagonist in comparison to the sleep restriction.

In the case of vermis, the basal lower density of A_2A_ adenosine receptors and its reduction during sleep restriction can explain the remaining blood-brain barrier hyperpermeability to dextrans even with the treatment with the selective A_2A_ adenosine receptor antagonist. Here, we again suggest that other mechanisms, independent of adenosine may be participating, such as blood-brain barrier regulation by pro-inflammatory cytokines (as hypothesized in [33]). Indeed it has been shown that the cerebellum seems to be prone to high-fat diet induced neuroinflammation mediated by the endogenous synthesis of the inflammatory mediators monocyte chemo attractant protein-1 (MCP-1), interleukin 1β (IL-1β), tumor necrosis factor-α (TNFα), and cyclooxygenase-2 (COX-2) as compared to the cerebral cortex [36]. In the case of the infection with the attenuated rabies virus CVS-F3 it has been shown that increased cerebellar blood-brain barrier permeability is accompanied by increased local synthesis of TNFα, interferon γ (IFNγ), and intercellular adhesion molecule-1 (ICAM-1) and occurs earlier in the cerebellum than in the cerebral cortex [37]. Additionally, the absence of sleep loss effects on tight junction protein expression in the cerebellar vermis point to a different mechanism by which sleep loss increases blood-brain barrier permeability in that region, such as pinocytosis.

The increase of adenosine levels in the brain after sleep loss [7] may induce the activation of A_2A_ adenosine receptors in endothelial cells but also in neural and glial cells [38] here we showed that A_2A_ receptors are present in parenchymal brain cells including neurons and endothelial cells. We also report that sleep restriction induced overexpression of markers of reactive microglia (Iba-1) and astroglia (GFAP) and that the administration of SCH58261 reverted this effect. Microglia participate in innate immunity, and several physiological processes such as in neurodevelopment and structural plasticity [39]. Interestingly, chronic administration of an inhibitor of microglia activation, minocycline, prevents the buildup of sleep need in rodents, suggesting that microglia might have a role in sleep regulation [40]. Microglial cells express all the subtypes of adenosine receptors, A1, A_2A_, A_2B_, and A_3_; however, particular attention has been paid to A_2A_, considered to have a central role in neuroinflammation [41]. In this way, the increase in Iba-1 expression may be related to the activation of microglia by adenosine acting on A_2A_ receptor during sleep restriction. On the other hand, astrocyte-derived adenosine is a candidate molecule involved in the cognitive deficits following sleep loss particularly at the hippocampal level [42, 43] and may have an important role in regulating blood-brain barrier permeability. Moreover, activation of A_2A_ receptor in astrocytes is related to activation of inflammatory status in several neuropathologies [44, 45]. In any case, gliosis is associated with blood-brain barrier disruption because the release of several inflammatory mediators [46–49]; however, it is also possible that glial cell activation may precede and even modify blood-brain barrier permeability, as shown after LPS administration [50].

**Fig 10:**
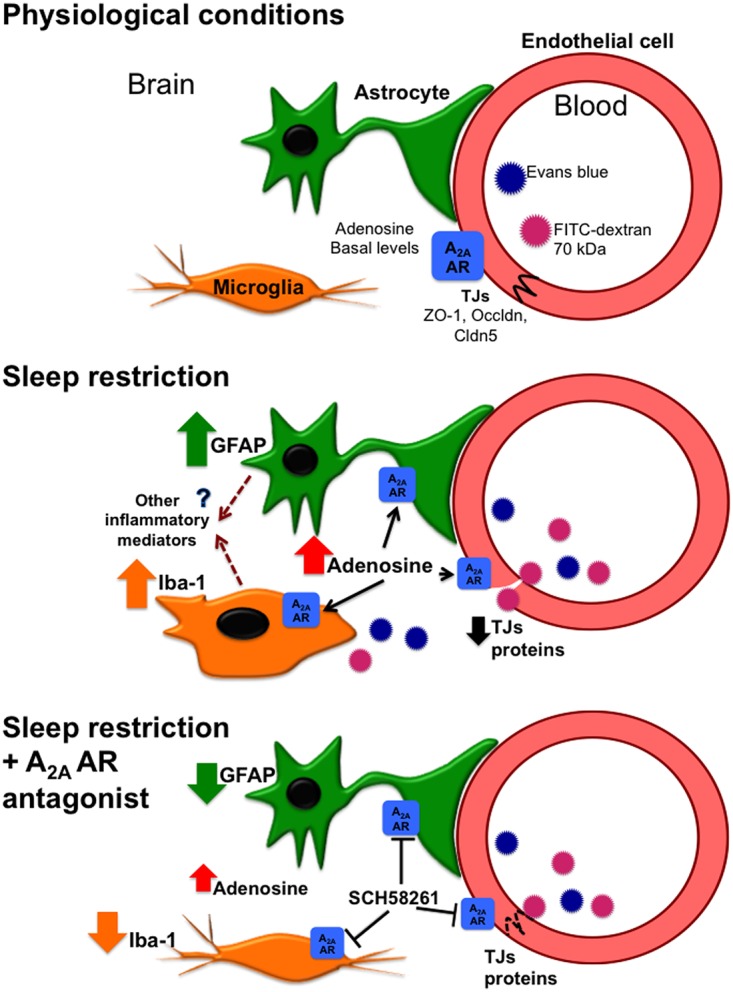
Blood-brain barrier regulation by adenosine during sleep restriction. Under physiological conditions adenosine modulates cerebral blood-flow and is involved in neurophysiological events including sleep regulation. Prolonged waking increases adenosine levels in the central nervous system. Sleep restriction induces gliosis, characterized by the overexpression of Iba-1 and GFAP markers, probably by the activation of A_2A_ adenosine receptor in the microglial and astroglial cells. The activation of glial cells may induce the release of other inflammatory mediators, eg. the pro-inflammatory cytokines IL-6 and TNF-α, which conjointly with adenosine may contribute to blood-brain barrier modulation during sleep loss. In endothelial cells, A_2A_ adenosine receptor activation promotes the decrease in tight junction protein expression, such as claudin-5 (Cldn5), occludin (Occldn) and Zonula occludens (ZO)-1. These changes may ensue blood-brain barrier hyperpermeability to molecules with high molecular weight such as 70-kDa FITC-dextran and Evans blue, and are rapidly reverted using a selective A_2A_ adenosine receptor antagonist such as SCH58261.

This study is the first to investigate A_2A_ adenosine receptor role in the regulation of blood-brain barrier during sleep restriction. We propose that changes in blood-brain barrier permeability contribute to many pathophysiological processes in the brain of subjects with sleep restriction (Fig 10). Those effects may be mediated by adenosine signaling that involves A_2A_ receptor activation and the regional differences in susceptibility may depend on adenosine receptor distribution and up-/down-regulation during sleep loss. More studies are needed to corroborate this hypothesis or explain this phenomenon taking into account that we evaluated the expression of A_2A_ adenosine receptor in specific brain regions but not in specific cellular types. It is probable that each cellular element of the blood-brain barrier, namely endothelial cells, pericytes, or astrocytes, may contribute to this phenomenon because of its potential adenosine-mediated signaling (for review see [12]). Indeed, it has been shown that during 12 hours of sleep deprivation astrocyte-derived adenosine is essential to alter network patterns of electrical activity in the frontal cortex [51]. Therefore, astrocytes may be playing a key role in regulating blood-brain barrier permeability during sleep loss through the release of adenosine and activation of A_2A_ adenosine receptors in brain endothelial cells; however *in vitro* assays are needed to clarify the role astrocyte-derived adenosine in regulating blood-brain barrier in sleep-restricted animals.

The findings here reported are relevant to consider the possible impact of chronic low-grade neuroinflammation in the development or exacerbation of neuropathologies associated with sleep deficiency. It may also contribute to generate knowledge about regulation of blood-brain barrier permeability using sleep restriction as a non-pathological model. In this way, sleep loss may promote temporal blood-brain barrier opening to allow the passage from blood-to-brain of molecules with potential therapeutic effects with a high regional specificity.

## Supporting Information

S1 Fig Sleep restriction increases the expression of the adherens junction protein E-cadherin in the hippocampus. At the top, representative western blot of the expression of E-cadherin in the cortex, hippocampus, basal nuclei, and cerebellar vermis. Graphs show the relative optical density of E-cadherin expression in the following groups: control plus DMSO (Con), sleep restriction plus DMSO (SR) and sleep restriction plus SCH58261 at 0.1mg/kg (SR+SCH). GAPDH was used for normalization. Mean ± s.e.m. Two-way ANOVA test *p<0.05 as compared to the control group.
(TIF)

S2 Fig Adenosine-synthesizing enzyme (CD73) expression remains unchanged despite chronic sleep loss. At the top, representative western blot of the expression of CD73 in the cortex, hippocampus, basal nuclei, and cerebellar vermis. Graphs show the relative optical density of CD73 in the following groups: control plus DMSO (Con) and sleep restriction plus DMSO (SR). GAPDH was used for normalization. Mean ± s.e.m.
(TIF)
