# Supplementary figures and images for: A2A Adenosine Receptor Antagonism Reverts the Blood-Brain Barrier Dysfunction Induced by Sleep Restriction
Source: PLoS One. 2016 Nov 28;11(11):e0167236. doi: 10.1371/journal.pone.0167236 (PMC5125701; doi:10.1371/journal.pone.0167236)

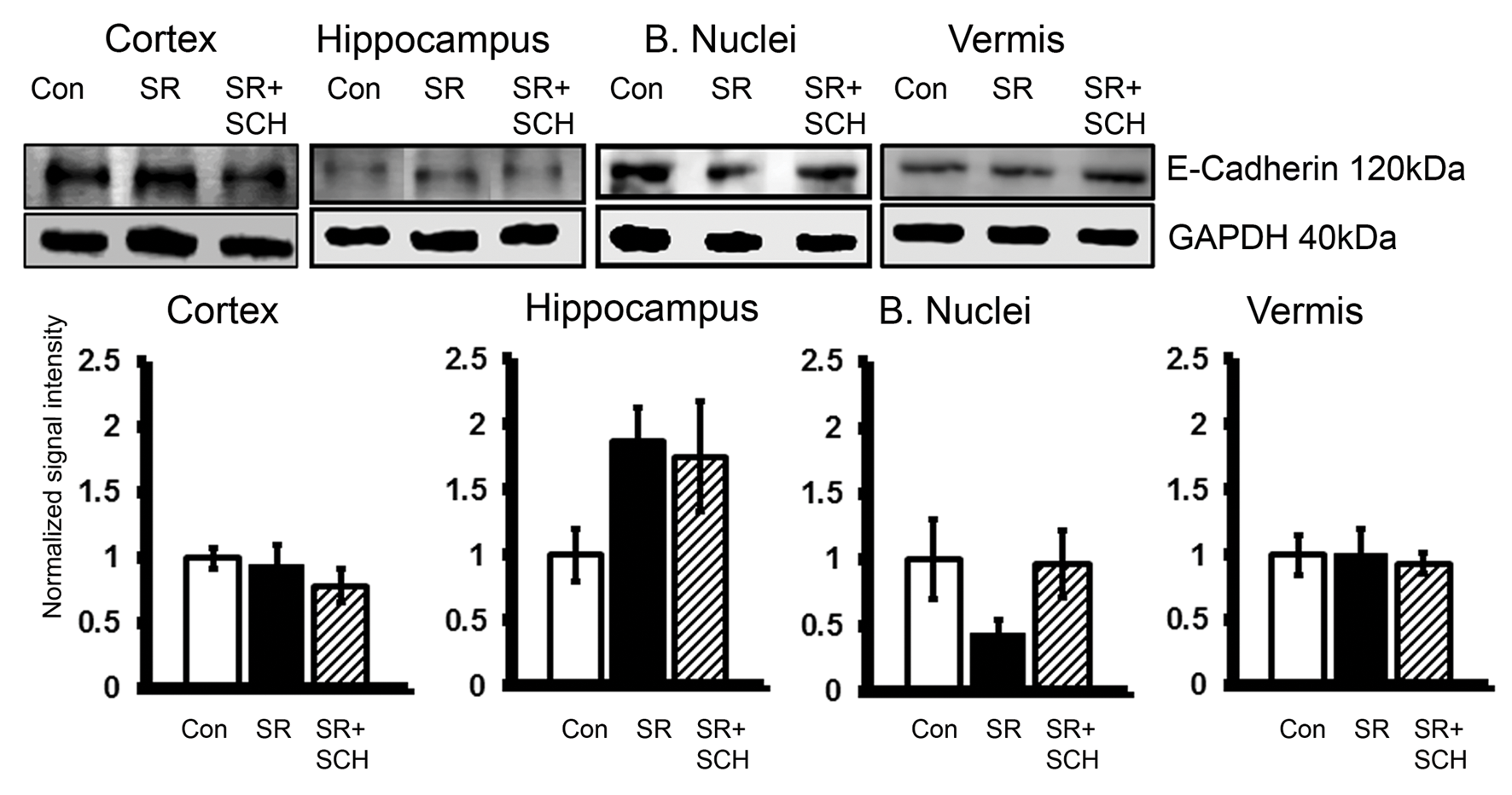

Supplement: S1 Fig — At the top, representative western blot of the expression of E-cadherin in the cortex, hippocampus, basal nuclei, and cerebellar vermis. Graphs show the relative optical density of E-cadherin expression in the following groups: control plus DMSO (Con), sleep restriction plus DMSO (SR) and sleep restriction plus SCH58261 at 0.1mg/kg (SR+SCH). GAPDH was used for normalization. Mean ± s.e.m. Two-way ANOVA test *p<0.05 as compared to the control group. (TIF) [file pone.0167236.s001.tif]

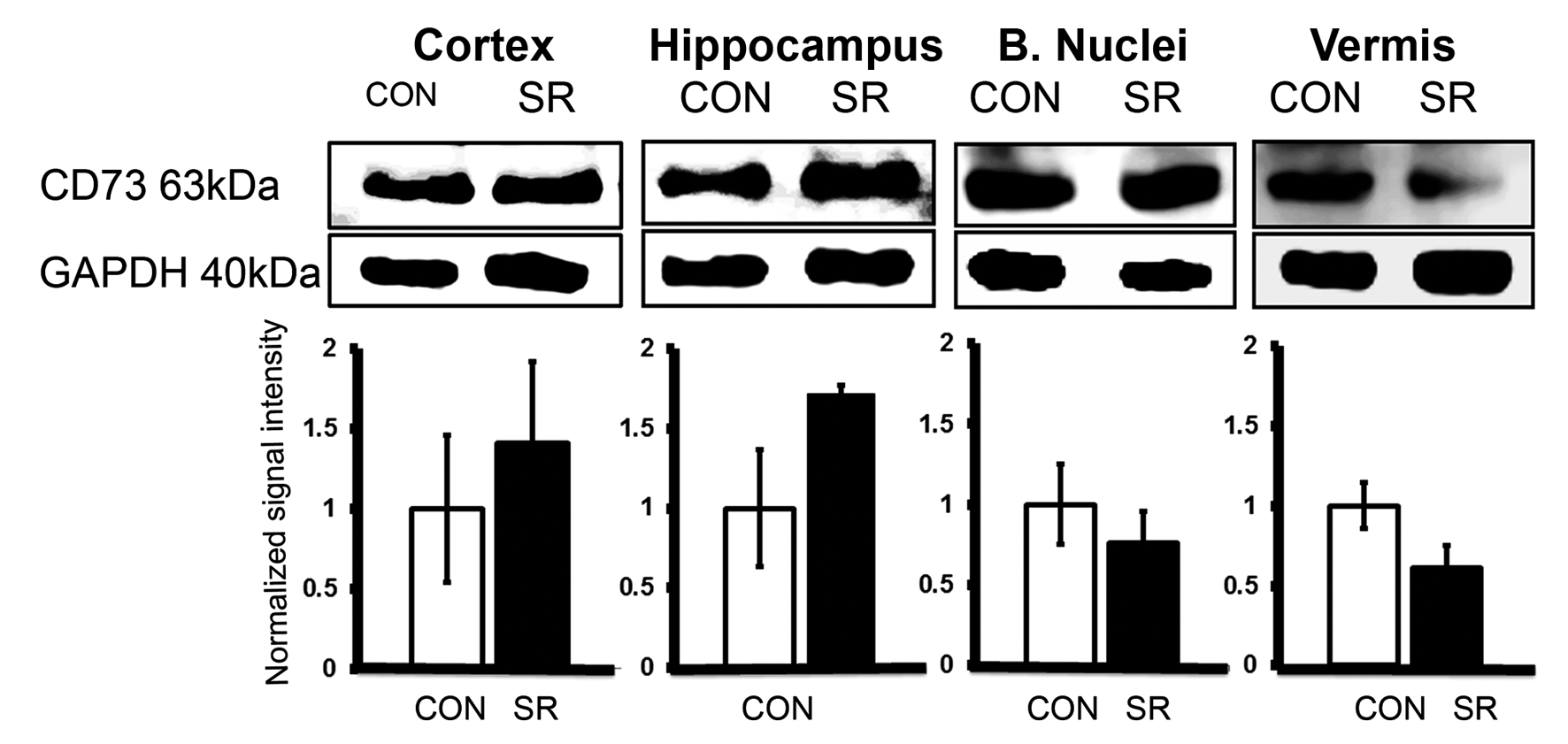

Supplement: S2 Fig — At the top, representative western blot of the expression of CD73 in the cortex, hippocampus, basal nuclei, and cerebellar vermis. Graphs show the relative optical density of CD73 in the following groups: control plus DMSO (Con) and sleep restriction plus DMSO (SR). GAPDH was used for normalization. Mean ± s.e.m. (TIF) [file pone.0167236.s002.tif]
